# Adverse childhood experiences and impact on quality of life in adulthood: development and validation of a short difficult childhood questionnaire in a large population-based health survey

**DOI:** 10.1007/s11136-021-02761-0

**Published:** 2021-02-03

**Authors:** John-Kåre Vederhus, Christine Timko, Siri Håvås Haugland

**Affiliations:** 1 Addiction Unit, Sørlandet Hospital, P.b. 416, 4604 Kristiansand, Norway; 2 Center for Innovation to Implementation, Veterans Affairs Health Care System and Stanford University School of Medicine, Palo Alto, CA USA; 3 Department of Psychosocial Health, University of Agder, Grimstad, Norway

**Keywords:** Adverse childhood experiences, Quality of life, Parental alcohol use, Mental health, Confirmatory factor analysis

## Abstract

**Purpose:**

A short adverse childhood experiences (ACEs) measure is needed with non-intrusive items that include subjective evaluations of childhood. We validated a short Difficult Childhood Questionnaire (DCQ) that assesses ACEs using personal perceptions of events.

**Methods:**

The study relied on 2019 data from a representative survey (*N* = 28,047) in Norway. We examined the DCQ’s factor structure, internal consistency, and discriminant validity in a multi-group confirmatory factor analysis. As a group variable, we used whether the respondent had the ACE of parental alcohol use disorder (adult children of alcoholics; ACOA). To assess the DCQ’s convergent validity, we used latent regression analysis with adulthood quality of life (QoL) as the outcome and mental distress and loneliness as potential mediators.

**Results:**

The DCQ’s latent mean was 0.86 (95% CI 0.82–0.90, *p* < 0.001) higher in the ACOA versus the non-ACOA group. The effect size suggested a large magnitude of this difference. The DCQ score was negatively associated with QoL and positively associated with mental distress and loneliness. For the score’s QoL effect [− 0.84 (95% CI − 0.87 to − 0.80, *p* < 0.001)], − 0.80 was indirect, and − 0.04 was direct. Thus, most of the association of DCQ with QoL occurred via mediators.

**Conclusions:**

The results confirmed the DCQ’s discriminant and convergent validity and highlight this tool as an empirically supported approach to assess ACEs. Because of its brevity and psychometric strengths, the DCQ is useful for research and likely suited to mental health treatment settings.

## Plain English Summary

A short measure of adverse childhood experiences with non-intrusive items is needed for assessing a person’s subjective evaluation of childhood. This study validated a short Difficult Childhood Questionnaire (DCQ) with data from a large, Norwegian public health survey (*N* = 28,047) in 2019. The DCQ discriminated well between a group with the adverse experience of having parents with an alcohol use disorder (adult children of alcoholics; ACOA) and a group without this experience. The ACOA group had DCQ scores that were 0.86 higher than the unexposed group, which was deemed a substantial difference. We also examined the relationship between the DCQ and adulthood quality of life (QoL), and the DCQ score was associated with a direct but rather weak negative influence on QoL, along with increased mental distress and loneliness. When including the mediated effects via mental distress and loneliness, the DCQ score led to an almost full point (− 0.84) worse QoL score, with − 0.80 representing indirect effects. Thus, most of the QoL effect associated with the DCQ score could trace to these potential aftermaths of adverse experiences in childhood, rather than being directly represented by the DCQ score itself. Because it is brief and potentially amenable for people seeking mental health treatment, the DCQ would likely be well suited to clinical settings. Its psychometric strengths render it useful for research purposes.

## Background

 Childhood adversities are prevalent [1–3], and no matter how they are measured, the association between these experiences and poor adult physical and mental health has been repeatedly confirmed [3–5]. More than 25% of cases of both anxiety and depression in Europe can be attributed to adverse childhood experiences (ACEs) [1], and a U.S. study found that patients with mood disorders were more likely to have a history of ACEs compared with the general population [6]. The relationship between ACEs and poor mental health is therefore recognized as an important focus area globally for prevention of non-communicable diseases [7]. Given these associations, as would be expected, ACEs are negatively associated with general and overarching concepts of health, such as quality of life (QoL) and well-being [8, 9].

One way that ACEs influence health is through social relationships [10, 11]. A stable and trustful environment helps a child build positive views of self and others and develop positive expectations regarding close ties [12]. Insecurity about relationships with important others limits trust in the world, leading to pessimistic views regarding social interactions [12]. Social connection to others is a powerful predictor of health and well-being [13, 14]. Conversely, lack of social connection, such as social isolation and loneliness, enhances risk for negative health outcomes and mortality [12, 15, 16].

Identifying adults with childhood experiences that could have affected their current health and well-being is relevant in clinical settings and from a public health perspective. Typical measures focus on parameters such as *types* of experience (e.g., family dysfunction, emotional/physical abuse, sexual abuse) and parameters for *quantifying* the experience (e.g., frequency of traumatic events, severity of the adverse experience). In many cases, this approach has led to instruments [17] with a length that may prevent inclusion of this topic in larger health surveys and records. Compared with the suggested 3-item scale in the present article, we note that the shortest reflective measure in a previous review of measurements in this field had 22 items [17].

The original and most common way to calculate ACE scores is to add up the number of “yes” responses to questions about specific maltreatment exposures before age 18 years and use the sum as a proxy for severity, sometimes called the ACE “dose” [18]. Previous findings indicate a “dose–response” relationship, and the cumulative impact on health problems is evident across the lifespan. For example, Chapman et al. found that the number of ACEs had a graded relationship to both lifetime and recent depressive disorders [19]. Having at least one ACE, such as a childhood experience of problematic alcohol use by a household member, also has been linked to the probability of having other adverse childhood experiences [20, 21].

The path from childhood adversities to later health problems is not inevitable, and children can become resilient adults, for example through available social support as emerging adults [22, 23]. For this reason, one helpful approach may be to use a paradigm with enhanced weight on resilience and ways of coping with an adverse experience. With such a paradigm, reflective items, such as self-perceived severity, might be better choices for inclusion rather than using only the sum of formative (objective) indicators, i.e., whether certain adverse experiences happened. The list of possible ACEs with potential to be traumatic for children (e.g., peer rejection and bullying) is much longer than the original ACE questionnaire list, yet these experiences would not be counted with formative indicators, simply because they are not mentioned [24].

A known problem in clinical settings is that patients may be reluctant to reveal that they had traumatic childhood experiences because of feelings of shame or unpreparedness when queried (for example, when a trauma has been more or less encapsulated), with a consequent risk of disrupting the relationship with health professionals [25]. Even simply screening for ACEs can potentially feel intrusive, raise discomfort, and add to a sense of stigma for the patient [24]. Direct and blunt questions about sexual abuse, for example, may then be non-therapeutic (e.g., “Did an adult or person at least 5 years older ever actually have oral, anal, or vaginal intercourse with you?) [26]. It would be sensible to use more global and generally worded items to avoid awkwardness and risk of stigmatization.

## Objectives

A brief ACE measure is needed that uses non-intrusive items that include subjective evaluations of the experiences. Accordingly, the aim of this study was to validate a short Difficult Childhood Questionnaire (DCQ) that assesses ACEs using non-stigmatizing items and personal perceptions of events. We examined the DCQ’s factor structure, internal consistency, and discriminant validity in a multi-group confirmatory factor analysis (MGCFA). We hypothesized that a group that had experienced a typical ACE (exposure to parental alcohol problems) would score higher on the DCQ than those who had not had this experience. Finally, we examined the convergent validity of the DCQ and hypothesized that it would be positively associated with mental distress and loneliness and negatively associated with QoL.

## Methods

### Participants and procedures

During autumn 2019, a representative sample of inhabitants (*N* = 61,611) from 30 municipalities in Southern Norway aged 18 or older were invited to complete an online survey with questions related to health, well-being, childhood, living conditions, local environments, accidents, and injuries. Invitations were based on randomly selected participants drawn from the Norwegian Population Registry of inhabitants in Southern Norway, and contact information (e-mail or telephone number) was retrieved from the contact registry from the Agency for Public Management and eGovernment (Difi). An online consent was provided as the participants answered the survey. Of the 61,611 invited respondents, a total of 28,047 completed the questionnaire, for a response rate of 45.5%. The study had a cross-sectional design with retrospective assessment of childhood experiences, as described below.

### Measures

*DCQ*. The DCQ was developed for and used in a large Norwegian public health study (the HUNT study). In HUNT 3, the third wave of this study, a single item was used (Q3 in Table 1) that allowed respondents to give a general evaluation of their childhood. The results based on this query showed strong relationships between perceiving childhood as difficult and a range of health-related outcomes [27]. For the fourth wave of the study (HUNT 4), the HUNT team thus sought to place greater focus on the theme of childhood difficulties. The overall questionnaire is quite long so that any addition had to be brief, preferably no more than two extra questions. Based on what was identified as the most important concepts, a question about communication and conflict level in the family (Q1 in Table 1) was included, as was a question about childhood trauma (Q2 in Table 1). To avoid potential embarrassment, the latter question was formulated indirectly to assess whether respondents struggled with difficult memories about adverse experiences during childhood because of loss, violence, or abuse. The items were scored on a 5-point ordinal scale (from “not at all” to “very much”), and higher scores represented greater perceived difficulties in childhood. The overall HUNT 4 survey was pilot tested in the municipality of Selbu. Respondents (*N* = 31) provided written comments to the questions, and six of these respondents were interviewed in detail by phone. A special focus was whether the questions were comprehensible and whether the respondents considered them to be inappropriate or too sensitive in nature. The items in the DCQ seemed to work well, and the pilot respondents offered no negative opinions.

**Table 1:** The questions of the DCQ

Item	Questions:
Q1	Var det mye krangling, uro, konflikter eller vanskelig kommunikasjon i barndomshjemmet?
	Translation: Did you grow up in a home marked by arguments, tension, conflicts, or poor communication?
Q2	Sliter du med vonde minner fra oppveksten pga. tap, svik, vanskjøtsel, vold, mishandling eller misbruk?
	Translation: Do you struggle with childhood memories of loss, violence, or being let down, abandoned, neglected, maltreated, or abused?
Q3	Når du tenker på barndommen/oppveksten din, vil du beskrive den som?
	Translation: When you think about your childhood, how would you describe it?

Response categories

Q1 and Q2: to a very great extent/ to a great extent/ to a limited extent/ to a very limited extent/ not at all (responses were reverse coded, so that higher scores indicate greater problems)

Q3: very good/ good/ average/ difficult/ very difficult

*SCL-5.* To measure mental distress, we used a short version of the Hopkins Symptom Checklist, the SCL-5 [28]. The SCL-5 performs almost as well as more extended versions (SCL-10 and SCL-25), and the reliability of the scale was excellent in the Norwegian validation study (Cronbach’s alpha = 0.87) [29]. Participants reported whether at any time in the previous week they had felt sad or depressed, hopeless about the future, tense or keyed up, constantly fearful and anxious, or worried. Response categories are “not bothered,” “a little bothered,” “bothered quite a lot,” and “extremely bothered,” scored 1–4. Higher scores indicate greater mental distress.

*Loneliness scale:* The Three-Item Loneliness Scale is a short version of the UCLA Loneliness Scale [30]. In a validation study, the reliability of the scale was acceptable (Cronbach’s alpha = 0.72) and appeared to measure overall loneliness quite well. This scale contains questions about how often the respondent feels a lack of companionship, left out, and isolated from others. The scale was developed for use in telephone surveys and has a simplified set of response categories. In the present online survey, the 5-point response categories ranged from “never” to “very often,” and higher scores indicate greater loneliness.

*CAST-6.* The questionnaire included the CAST-6 scale [31], used to assess whether or not participants perceived their parents’ alcohol consumption as problematic. CAST-6 is a brief version of the 30-item Children of Alcoholics Screening Test (CAST) [32] and has demonstrated high internal consistency and test–retest reliability. In a previous validation study, the Cronbach’s alpha ranged from 0.86 to 0.92 for students and patients being treated for substance use disorder [31]. The validity of the CAST-6 has been confirmed in adult populations [31, 33] and among adolescents [34]. Items include questions about whether respondents have ever thought of one of their parents as having a drinking problem, encouraged one of their parents to quit drinking, argued with a drinking parent, heard parents fight when drinking, or felt like hiding or emptying a parent’s bottle of alcohol, and whether they had wished their parent would stop drinking. Response categories for the six items are yes/no. Responses are summed (scale 0–6), and for this study, a score of ≥ 3 was defined as a parental alcohol problem. The original measure used the term “adult children of alcoholics” (ACOA). For simplicity, we also use this term, although we note that “alcoholics” is now outdated, and first-person language is preferred (“children of parents who had problematic alcohol use”) [35].

*QoL.* We used three items representing subjective QoL, all of which have been suggested as part of a “minimum” list for measuring QoL in national public health surveys in Norway [36]. The items represent three dimensions of subjective QoL: cognitive (satisfaction with life), affective (positive feelings such as happiness), and eudaimonic (whether life is perceived as meaningful) [37, 38]. Conceptually, the first two items address classical subjective well-being (hedonic). The third item builds on the Aristotelian view that a “good life” comprises not only desire fulfillment but also whether a person lives up to their perceived potential or in accordance with their perceived purpose in life. The items are rated on 0–10-point scales, with higher scores representing more satisfaction, happiness, and perceived meaningfulness in life. To our knowledge, these items have not been validated as an expression for QoL in a latent variable analysis before using a structural equation modeling (SEM) framework. Because we used this scale as an outcome measure in our mediation analysis and associations are bi-directional, our findings indirectly assessed the convergent validity for the QoL scale. In other words, the analysis indicated whether the associations of QoL with the DCQ scores and the mediators were in the expected direction. We also report the internal consistency of the scale.

### Statistical analyses

Descriptive statistics are used to show sample characteristics. The constructs used in the following analyses were handled as latent variables in a SEM framework, so that the questions were modeled as reflective indicators of their respective constructs [39]. Mplus version 8.4 was used for the analyses. Because of the ordinal nature of the DCQ, we used the maximum likelihood estimation with robust standard errors (MLR) and report standardized factor loadings (beta = *β*) [40]. The default procedure in Mplus, full information maximum likelihood, handled missing values. The significance level was set at *p* < 0.05. The root mean square error of approximation (RMSEA) and the comparative fit index (CFI) were used to assess goodness of fit, with a cut-off value for a good model fit of 0.05 or less for RMSEA and 0.95 or higher for CFI [41, 42].

We assessed the discriminant validity of the DCQ with an MGCFA, examining whether the DCQ discriminated between a group with expected higher negative childhood experiences (ACOA) versus a group without these experiences (non-ACOA). To legitimately be able to compare means between the two groups, we first examined whether the DCQ measured the construct similarly in the two groups to ensure that the construct was invariant (equivalent) across groups [43]. First, we established a configural (baseline) model. The reliability of the scale is reported with the composite reliability (CR) value [44], and a CR value of > 0.70 is considered acceptable. For convenience, we also added McDonald’s omega coefficient [45].

We then implemented a simultaneous MGCFA with increasing equality constraints on measurement parameters to examine if the scale had strong measurement invariance (scalar equivalence) across groups. Scalar equivalence implies that the scales have the same factor structure (configural equivalence) and equivalent factor loadings (metric equivalence) across groups, as well as invariant intercepts, with equivalent origin on the scales in the different groups [46]. If scalar invariance is not achieved, intercepts of the non-invariant items could be freed to assume partial scalar equivalence, which is still considered sufficient for comparing latent means if at least two items are metrically invariant per factor [47]. The testing implies that the more restricted models are compared with the less restricted models, and chi-square differences between the nested models illustrate whether differences in the chi-square value (∆*χ*2) relative to the change in degrees of freedom (∆df) are significant [40]. A non-significant ∆*χ*2 value indicates that constraining does not worsen the fit of the model and that the null hypothesis of measurement invariance can be retained [48]. However, Cheung and Rensvold found in a simulation study that the chi-square test might be too conservative, especially in large samples, running the risk of being too likely to deem a measure as non-invariant [49, 50]. They recommended using the change in CFI (∆CFI), which sample size does not affect. A value of ΔCFI less than or equal to − 0.01 indicates that the null hypothesis of invariance should not be rejected [49]. Latent mean differences are reported as unstandardized β values with 95% confidence intervals (CIs).

We assessed the convergent validity of the DCQ by examining whether the score was related in the expected direction with constructs from the literature that are known to correlate with adverse childhood experiences (mental distress, loneliness, and QoL). Results of this latent regression analysis are reported with unstandardized *β* and 95% CI values to facilitate interpretation of the associations between constructs. The *R* square (*R*^2^) value was used to assess the percentage of the variation in the latent constructs explained by the model.

## Results

### Participant characteristics

Most of the participants (76%) were between 25 and 66 years old, slightly more than one-half (53%) were female, about one-half had a higher educational level (at least a bachelor’s degree), and 8 in 10 lived with a spouse or partner (Table 2). According to the CAST-6, about one in six was defined as a child of a parent(s) who had a problematic alcohol use (ACOA).

**Table 2:** Participant characteristics (*N* = 28,047)

	N (%)
Age group	
18–24 years	3169 (11)
25–44 years	9180 (33)
45–66 years	12,026 (43)
67–79 years	3372 (12)
80 + years	300 (1)
Female sex	14,925 (53)
Educational level (*n* = 27,923)	
Primary and secondary school (up to 10 years of education)	3333 (12)
High school (up to 13 years of education)	11,088 (40)
University college or university (bachelor’s degree or higher)	13,502 (48)
Living with a spouse or partner (*n* = 27,977)	21,893 (78)
Child of parent(s) with a problematic alcohol use (*n* = 27,895)	4346 (16)

### Internal consistency and discriminant validity of the DCQ

**Fig. 1:**
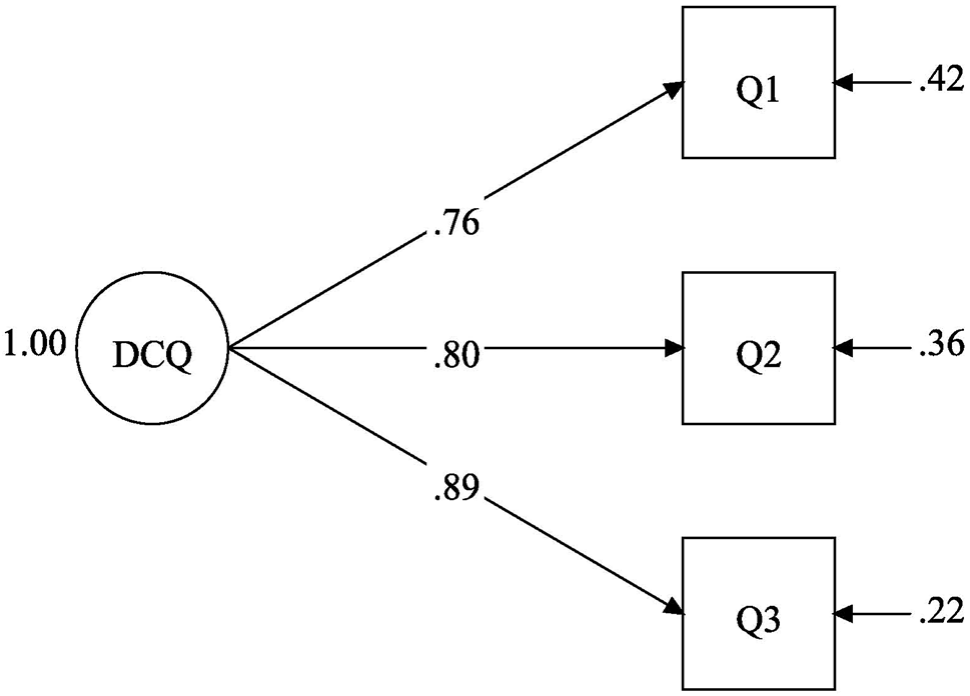
Baseline model for the difficult childhood questionnaire (DCQ). The model was similar across groups, and the figure shows the standardized factor loadings and residual (error) variances for the adult children of alcoholics (ACOA) group. Q1–Q3 refers to the indicators (questions) shown in Table 1

The covariance coverage of the indicators used in the following analyses were good, above 98.8% in all cases. The first step was specification of the baseline model of the DCQ for each group (Fig. 1). This one-factor solution had “perfect” goodness-of-fit values (RMSEA = 0.00 and CFI = 1.00). Although a model with three indicators is “just-identified” and goodness-of-fit evaluation does not apply, the model can still be evaluated in terms of interpretability and strength of its parameter estimates (e.g., magnitude of factor loadings) [51]. The model had factor loadings from 0.69 to 0.89 in both groups, and no error correlations emerged. The baseline model for the ACOA group is shown in Fig. 1. The reliability of the scale was excellent (CR and omega coefficient = 0.86).

The MGCFAs specifying the *χ*2 and CFI values for the configural, metric, and scalar models are shown in Table 3. In case of the metric and scalar models, these values have positive degrees of freedom, as more restrictions were set on the model. Thus, they are over-identified models, and comparison between them is legitimate [51]. The ∆*χ*2s between the nested models were significant as expected because of the large number of participants; thus, further investigation was based on ∆CFIs. The scalar model had a 0.03 lower CFI value compared with the metric model, and its RMSEA was too high, indicating a problem. The modification indices indicated that the intercept of Q1 (Table 1) was non-invariant and that the ∆*χ*2 would improve considerably if this intercept was freed. The final partial scalar model had excellent fit and was deemed as invariant because the ∆CFI was ≤  − 0.01 (Table 3), so latent means could be compared. The latent mean of the DCQ scale was 0.86 (95% CI 0.82–0.90, *p* < 0.001) higher in the ACOA group versus the non-ACOA group. To examine the magnitude of this value, we calculated an effect size *d* for the latent mean differences following the procedure proposed by Hancock [52]. The effect size *d* for the ACOA versus the non-ACOA group was 1.15 (see calculation in additional file). According to interpretative guidelines, 0.2, 0.5, and 0.8 are cut-off values for small, medium, and large effect sizes of latent means, respectively, which indicates a large effect size of differences between groups [52].

**Table 3:** Multigroup confirmatory factor analysis results of the measurement invariance tests across the two groups: ACOA (*n* = 4346) and non-ACOA (*n* = 23,549)

	χ^2^	*df*	RMSEA	CFI
Configural model	0.00	0	0.00	1.00
Metric model	51	2	0.04	1.00
Scalar model	618	4	0.11	0.97
Partial scalar model	100	3	0.05	1.00

Note: 152 had missing data on all variables used in the analysis

*ACOA* Adult children of alcoholics; *CFI* Comparative fit index; *RMSEA* Root mean square error of approximation

### Convergent validity

**Fig. 2:**
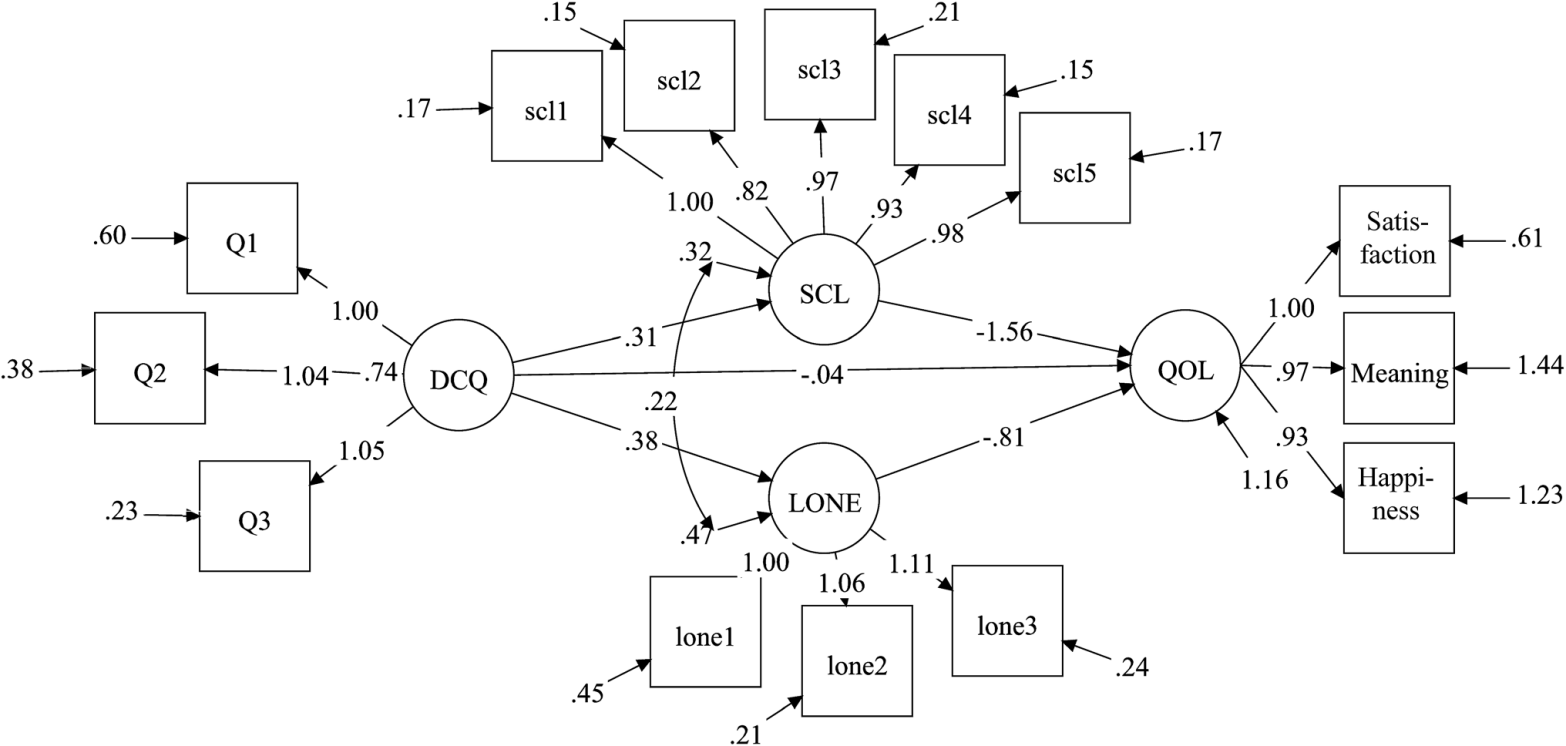
Latent regression analysis showing the association of Difficult Childhood Questionnaire (DCQ) scores with adulthood quality of life (QoL) and the effect of two mediators, mental distress (SCL) and loneliness (Lone). The figure shows the measurement and the structural model with unstandardized factor loadings

Continuing to the convergent validity analyses, we tested the relationship between the DCQ and adulthood QoL in a simultaneous mediation analysis that included mental distress (SCL-5) and loneliness as mediating constructs. The DCQ scores had a direct negative association with QoL (*β* =  − 0.04, 95% CI − 0.07 to − 0.02, *p* = 0.001; Fig. 2). The DCQ scores were associated in the expected direction with both mental distress (*β* = 0.31, 95% CI 0.30–0.33, *p* < 0.001) and loneliness (*β* = 0.38, 95% CI 0.36–0.39, *p* < 0.001), i.e., the level of mental distress and loneliness increased when the DCQ score increased. In turn, mental distress and loneliness had a substantial negative influence on QoL (*β* =  − 1.56 and *β* =  − 0.81, respectively; Fig. 2). The specific indirect effect of the DCQ score in association with QoL (shown only in the Mplus output, not in the figure) via mental distress was *β* − 0.49 (95% CI − 0.51 to − 0.46, *p* < 0.001). The specific indirect effect of this score in association with QoL via loneliness was *β* − 0.31 (95% CI − 0.33 to − 0.29, *p* < 0.001). Thus, the DCQ accounted for 31% of the influence of the mental distress construct (− 0.49/ − 1.56) on QoL and 38% of influence of the loneliness construct on QoL (− 0.31/ − 0.81). The total influence of the DCQ score in association with QoL was − 0.84 (95% CI − 0.87 to − 0.80, *p* < 0.001), and of this value, − 0.80 was indirect and − 0.04 direct. The model as a whole had excellent goodness-of-fit indices (RMSEA = 0.05 and CFI = 0.98) and explained 18% of the variance in mental distress, 19% of the variance in loneliness, and 65% of the variance in QoL (*R*^2^). The reliability of the QoL scale was excellent (CR and omega coefficient = 0.90).

## Discussion

This analysis confirmed the discriminant validity of the DCQ, with a group who had experienced a prevalent ACE reporting substantially higher scores on the DCQ than their counterparts without this experience (parental problematic alcohol use). The convergent validity of the DCQ was also confirmed, showing associations in the expected direction with tools that measure potential sequela of ACEs: loneliness, adverse mental health outcomes, and decreased QoL. The main association of the DCQ score with QoL could be attributable to the mediating constructs of mental distress and loneliness.

The findings support the hypothesis that the DCQ could be a valid and useful measure in large public health surveys. Of note, the wording of the DCQ assesses ACEs indirectly, with phrasing in terms of difficult memories. The DCQ also lumps several possible ACEs together in the same question, which may make respondents feel less exposed than the direct and blunt approach used in the original ACE measure. Thus, the DCQ approach will likely be perceived as less intrusive and stigmatizing and more in line with the relationship requirements of a modern clinical practice paradigm. In clinical settings, the DCQ could serve as a sensitive first step to map whether childhood adversities may be an issue, while still attending to the development of a trusting provider–patient relationship [25].

The original ACE questionnaire is still in use in several large-scale public health surveys, such as the Behavioral Risk Factor Surveillance System, conducted by the U.S. Centers for Disease Control and Prevention to collect data on health practices and behavioral risk factors among U.S. adults [53]. The original ACE questionnaire has even been launched online on various websites such as “Get your ACE score” or “Take the ACE quiz” [54, 55]. Although such sites typically feature a disclaimer about what the questionnaire does and does not mean, people who take the test are at risk of inferring an inevitable link between a higher score and later negative health consequences. The ACE score does not consider positive experiences in early life that could help build resilience and protect a child from the effects of trauma, and this omission is easy to overlook [54].

An important finding in the current work is that a higher DCQ score was associated with lower QoL, consistent with previous studies of ACEs and well-being [56]. However, in our mediation analysis, the direct negative association of DCQ score with QoL was rather modest based on a nominal evaluation: a one-unit increase in the 5-point DCQ scale accounted for a 0.04 decrease on the 10-point QoL scale. The negative effect association between DCQ score and QoL grew substantially when mental distress and loneliness, both possible residues of childhood adversities, were examined as mediating mechanisms: a one-unit increase in the DCQ led to a total 0.84 reduction in the QoL score when these indirect effects were added to the model, representing a quite strong negative influence on a 10-point scale. Thus, our model demonstrated that mental health and feelings of social isolation are conduits for childhood adversities to exert their influence on overall measures of health in adulthood, such as QoL.

We have not identified previous QoL studies examining mediation processes in a latent SEM framework, but our finding resembles reports from previous studies of ACEs and QoL. In a U.S. community-based study, the influence of ACE score on health-related QoL weakened when potential sequelae of ACEs (stress and sleep disturbances) were added to the model [57]. Indeed, the direct influence of ACEs on health-related QoL became non-significant when these mediators were added, which indicates total mediation [57]. Together, such findings support the notion that studies should not focus on the mere presence of ACEs but examine possible pathways that connect childhood adversities to health outcomes [58]. The question to address is whether childhood adversities resulting in health impairments depend on the proximal mechanisms at work. From a treatment perspective, the good news is that clinicians have effective interventions directed at the potential sequelae of ACEs, including post-traumatic stress disorder, depression, and substance use disorders. When adults with ACEs need help, they can receive support that includes existing effective remedies targeting these conditions [24].

## Methodological considerations

Among the strengths of this study is its large sample size drawn from a general population. The novelty of the current study is the development of a short instrument that includes reflective items. The methodology typically used in this field relies on a detailed mapping of specific experiences, which may be perceived as intrusive and stigmatizing and increase non-response. A review of the validity of retrospective reports of ACEs among adults has shown that such reports may include a substantial number of false negatives [59]. The DCQ used in our study can potentially reduce this under-reporting. However, retrospective bias, such as the possibility that resilient adults have made cognitive adjustments of their evaluations of childhood, cannot be avoided even with use of reflective indicators. Another limitation is that the general nature of our measure could limit the possibility of examining specific ACEs, such as whether differing types of maltreatment are differentially associated with adult health outcomes, e.g., depression [60].

## Conclusions

The DCQ offers an empirically supported method of assessing ACEs. It is well suited to clinical settings because it is brief and likely to be amenable to people seeking mental health treatment. Its brevity and psychometric strengths make it useful for research purposes ranging from large, epidemiological studies to intervention trials. Use of the DCQ in treatment and research settings should benefit people who continue to struggle with the aftermath of ACEs.

## Data Availability

The controller for this public health survey (NIPH) will deposit the data in a publicly available data repository: https://helsedata.no/en/. For more information, contact the first author.
